# Evaluation of the *Salmonella* type 3 secretion system (T3SS) as part of a protein production platform for space biology applications

**DOI:** 10.3389/fbioe.2025.1567596

**Published:** 2025-04-02

**Authors:** Min-Kyoung Kang, James Bevington, Danielle Tullman-Ercek

**Affiliations:** ^1^ Department of Chemical and Biological Engineering, Northwestern University, Evanston, IL, United States; ^2^ Anti-aging Bio Cell factory Regional Leading Research Center (ABC-RLRC), Gyeongsang National University, Jinju, Republic of Korea; ^3^ Interplanetary Exploration Institute Ltd., Sydney, Australia

**Keywords:** microgravity, *Salmonella*, type 3 secretion system (T3SS), protein secretion, synthetic biology

## Abstract

As interest in space exploration and *in situ* resource utilization grows, the potential to leverage synthetic biology and engineered microorganisms has garnered significant attention. Microorganisms provide a robust and efficient biological chassis to demonstrate the human blueprint for advancing space biology. However, progress toward these applications is hindered by the limited access to space-like environments and a lack of knowledge about how unique environmental factors affect relevant microbial systems. To address these issues, we evaluated the *Salmonella* Pathogenicity Island 1 (SPI-1) type Ⅲ secretion system (T3SS) as a protein production platform for space applications. Using a NASA-designed microgravity-simulating bioreactor system, we investigated the effects of simulated microgravity on cell growth, stress response, and protein secretion *via* SPI-1 T3SS. Our results demonstrated increased stress responses in cells grown under simulated microgravity. However, the SPI-1 T3SS maintained its ability to secrete proteins directly into the extracellular space in a single step under simulated microgravity, simplifying downstream purification processes. These findings suggest that the SPI-1 T3SS is a viable candidate for future space biology applications.

## 1 Introduction

Humans have an instinctive curiosity to explore unknown territory, including space, which in turn prompts the development of innovations that improve daily life (Comstock and Lockney, 2007). In addition, advances from space exploration technologies are often uniquely suited for *in situ* resource utilization (ISRU) in terrestrial contexts where resources and/or infrastructure are limited. Synthetic biology, which is a multidisciplinary field combining biology and engineering, is considered one of the key technologies for developing biomanufacturing with limited resources and for space applications (Menezes et al., 2015; Brooks and Alper, 2021; Tang et al., 2023). In particular, synthetic biology can be used to systematically, and modularly, engineer microbial hosts, which is seen as a powerful solution to address the challenges of space exploration and ISRU (Menezes et al., 2015; Santomartino et al., 2023).

The application and transfer of current synthetic biology technologies to space are hindered by the unique environmental conditions encountered beyond Earth, for which there are currently limited data sets. Understanding the effects of space environments on biological systems is imperative for future applications. The International Space Station (ISS) has served as the primary location for conducting space biology research, but its limited accessibility and high operational costs restrict more extensive research. Ground-based microgravity simulators, such as rotary cell culture systems (RCCS) with High Aspect Ratio Vessels (HARVs), were designed by researchers at NASA Johnson Space Center to build low-shear environments to simulate this aspect of the space environment (Schwarz and Wolf, 1991; Schwarz et al., 1992). This ground-based microgravity simulator system has been widely used to study the effects of microgravity on microbial hosts (Nickerson et al., 2003; Anken, 2013; Acres et al., 2021). Multiple studies have reported that microorganisms, including *Salmonella*, respond to microgravity by altering physiological processes, gene expression, and stress responses (Nickerson et al., 2000; Tucker et al., 2007; Wilson et al., 2007; Kim and Rhee, 2016; Yang et al., 2016; Senatore et al., 2020; Su et al., 2021).

Proteins are essential macromolecules which serve diverse biological functions across all organisms and have become indispensable components in a variety of industrial applications, ranging from degradative enzymes to advanced materials and therapeutics (Whitford, 2005; Ferrer-Miralles et al., 2009; Overton, 2014). To meet the increasing industrial demand for these multifunctional biomolecules, microbial expression systems have emerged as the dominant platforms for recombinant protein production. These systems have been continually optimized to enable efficient protein synthesis on an industrial scale (Ferrer-Miralles et al., 2009; Overton, 2014; Chen et al., 2021; Tepap et al., 2023). Potential applications of proteins extend beyond terrestrial environments to space, especially supporting ISRU and long-duration missions. The versatility of proteins could address a diverse range of challenges in extraterrestrial environments, from the biomanufacturing of replacement or novel materials, such as biopolymers and biomaterials for radioprotection and habitation (Ouchen et al., 2014; Jemison and Olabisi, 2021) to the development of targeted medical treatments including personalized medicine and regenerative medicine for damages caused by radiation (Pavez Loriè et al., 2021). Furthermore, establishing microbial protein production platforms in space could provide adaptable and resource-efficient biomanufacturing facilities that are important for future space missions (Averesch et al., 2023; Santomartino et al., 2023).

Bacterial protein secretion systems are promising technology to enable the modular, on-demand production of such useful recombinant proteins. Harnessing native bacterial secretion mechanisms could simplify protein production in space by eliminating the need for complicated cell lysis and downstream purification steps that otherwise hinder protein production from adoption for resource-limited situations (Reed and Chen, 2013; Burdette et al., 2018). Among the microbial strains extensively studied in synthetic biology, *Salmonella* species have gained attention for its applications in medicine and pharmaceuticals (Yoon, 2018; Yan et al., 2023) due to their tractability and their high genetic similarity to *Escherichia coli*, which enables many tools developed in the canonical Gram-negative model system to be successfully transferred with relative ease. The *Salmonella* Pathogenicity Island 1 (SPI-1) type Ⅲ secretion system (T3SS) is a highly efficient tool for recombinant protein secretion (Metcalf et al., 2014; Azam et al., 2016; Metcalf et al., 2016; Song et al., 2017; Burdette et al., 2021; Liang et al., 2023; Burdette et al., 2024). The T3SS, utilized by many Gram-negative bacteria, comprises a needle-like structure known as the injectisome which is natively used to translocate effector proteins from the cytosol directly into eukaryotic hosts, but also functions in the absence of such hosts to secrete heterologous proteins appended to appropriate N-terminal signal sequences in one step to the extracellular space (Green and Mecsas, 2016; Burdette et al., 2018; Lou et al., 2019; Burdette et al., 2024). The system is able to secrete a wide variety of protein cargo, including antibody fragments, enzymes (Metcalf et al., 2016), material-forming proteins (Widmaier et al., 2009; Azam et al., 2016), and growth factors (Burdette et al., 2024), across a range of sizes (up to ∼95 kDa) (Widmaier et al., 2009), many of which are able to fold and function following secretion to the media in bacterial culture (Metcalf et al., 2016), at reported titers of up to 140 mg/L (Glasgow et al., 2017). Thus, the SPI-1 T3SS shows promise as a key component of a bacterial platform for recombinant protein production in resource-limited environments like space. While traditional intracellular protein expression systems require extensive and specialized downstream processing steps, the SPI-1 T3SS confers up to 80% purity without any additional purification steps, with further purity to >90% following as few as one additional unit operation (Azam et al., 2016). Despite these advantages, recombinant protein production through the SPI-1 T3SS remains subject to environmental sensitivity, which is attributed to its intricate regulatory network (Tartera and Metcalf, 1993; Ellermeier et al., 2005; Golubeva et al., 2012; Song et al., 2017; Lou et al., 2019). Thus, it is unknown how its function may be affected by conditions of relevance to space environmental conditions.

In this study, we sought to investigate the effects of simulated microgravity on the SPI-1 T3SS as a potential protein production platform for space applications. We employed *Salmonella enterica* serovar Typhimurium ASTE13, a high-efficiency protein secretion strain (Song et al., 2017; Burdette et al., 2021; Liang et al., 2023). By comparing growth and stress responses under different gravitational conditions, we evaluated SPI-1 T3SS functionality through transcriptional activity and protein secretion experiments. Consistent with previous reports, we observed altered stress responses to simulated microgravity in this strain (Nickerson et al., 2003; Yang et al., 2016). Interestingly, the SPI-1 T3SS showed comparable secretion activity under both gravitational conditions, indicating its potential as a robust bio-device for future space missions.

## 2 Materials and methods

### 2.1 Strains and plasmids

*Salmonella enterica* serovar Typhimurium ASTE13 (ASTE13), a derivative of *S. enterica* serovar Typhimurium DW01 (Song et al., 2017; Burdette et al., 2021; Liang et al., 2023), or its derivatives were used in this study unless otherwise noted. All strains and plasmids used in this study are listed in Table 1.

**TABLE 1:** The list of strains and plasmids used in this study.

Name	Description	References
Strains		
ASTE13	*Salmonella enterica* Typhimurium ASTE13	Burdette et al. (2021)
WT-DH	ASTE13 harboring P_sicA_-DH	Widmaier et al. (2009)
Δ*prgI*-DH	*prgI* deleted ASTE13 harboring P_sicA_-DH	Burdette et al. (2024)
*sipC*:GFPmut2	genome integration of GFP next to the *sipC* in ASTE13	Burdette et al. (2021)
Plasmids		
P_sicA_-DH	*sicA* promoter, *sicP*, sptP-DH-2xFLAG-6xHis, colE1, Cmp^R^	Widmaier et al. (2009)

### 2.2 Growth conditions

ASTE13 was used to compare the growth and stress resistance at both gravitational conditions. A single colony of ASTE13 from a lysogeny broth (LB) agar plate was inoculated into LB-Lennox (L) media (10 g/L tryptone, 5 g/L yeast extract, and 5 g/L NaCl) and statically cultured overnight at 37°C. The overnight culture samples were diluted to an OD_600_ of 0.01 in LB-L and then transferred to the HARVs for the culture on the RCCS (10 mL autoclavable HARVs, RCCS-4D, Synthecon Inc., United States) at 37°C and 25 rpm in a humidified incubator.

### 2.3 Stress response and viability assays

After the 10-h cultivation in the HARVs, bacterial cultures were either kept untreated or exposed to one of the following three stress conditions. For the pH stress experiment, 1M of concentrated citrate buffer (pH 3.0) (Yang et al., 2016) was added to the culture, which was then held for 30 min at room temperature. For the heat stress experiment, cultures were placed in a water bath at 50°C for 30 min. For the bile salt stress experiment, 10% (w/v) of bile salt was added to the cell cultures for 30 min. Following the stress exposure, cell viabilities were analyzed using LIVE/DEAD staining followed by flow cytometry to compare the survival rate under stress conditions. The viability assay was carried out by following the manufacturer’s protocol for the LIVE/DEAD Bacterial Viability Kit (L7012, Thermo Fisher Scientific). Briefly, the cell cultures were adjusted by OD_670_of 0.03 and then diluted 100 times in PBS buffer. Then, 3 µL of the reagent mixture from the kit was added to each sample and incubated at room temperature for 15 min in the dark. After the incubation, the samples were transferred to a 96-well plate and analyzed by flow cytometry using an Attune NxT flow cytometer (Life Technologies) for fluorescence measurements. Data analysis was performed by FlowJo.

### 2.4 RCCS setup

Autoclavable HARVs were selected due to their ability to be re-used, enabling experimental consistency and replications. Prior to all experiments using the RCCS with HARVs, the vessel membranes were pre-wet and the HARVs were checked for contamination issues by filling 70% of the vessel volume with culture media followed by rotating at 25 rpm and 37°C overnight. For bacterial culture experiments using the RCCS with HARVs, the media pre-occupied vessels were emptied and then the diluted bacterial culture was transferred to, and completely filled, the HARVs to avoid bubble formation. This required maintaining a larger volume of diluted culture than was needed for the HARVs. Before attaching the HARVs to the RCCS, the HARVs were checked for the presence of any bubbles. If present, the remaining bubbles were removed using the syringe attached to the HARVs. The RCCS was placed at 37°C in a humidified incubator and the rotation speed of HARVs was set at 25 rpm unless otherwise noted. End-point sampling was used for all experiments to prevent any issues related to microgravity disruption.

### 2.5 *Salmonella* T3SS promoter activity assay

To assess promoter activity under different gravitational states, ASTE13 *sipC*:GFPmut2 was used. GFPmut2 acts as a reporter of P_sicA_ promoter activity (Burdette et al., 2021). This strain was streaked from a frozen stock, and a single colony was inoculated into 5 mL of LB media with 0.4% (w/v) glucose and cultured overnight at 225 rpm and 37°C. The overnight culture was diluted 100-fold in LB-L and then transferred to the HARVs and loaded onto the RCCS. The cultures were grown at 37°C and 25 rpm. As noted above, there was a single HARV culture used for each time point. Thus for each time point, a vessel was removed from the RCCS and samples were withdrawn from the HARV. These were diluted to an OD_600_ of 0.01 in PBS with 1 mg/mL kanamycin and transferred to a 96-well plate, which was stored at 4°C overnight. The next day, the cells were analyzed by flow cytometry using the Attune NxT flow cytometer. Data was analyzed using FlowJo 10.5.3.

### 2.6 *Salmonella* protein secretion

ASTE13 and ASTE13 Δ*prgI* strains were transformed with the plasmid harboring P_sicA_-DH (Widmaier et al., 2009), which encodes for the catalytic domain of the human intersectin-1 protein, to create the strains referred to herein as WT-DH and Δ*prgI*-DH, respectively (Table 1). A single colony was inoculated into 5 mL of LB-L media with chloramphenicol (34 μg/mL) and cultured overnight at 225 rpm and 37°C. The overnight culture was diluted 100-fold into LB-L with chloramphenicol (34 μg/mL) and transferred to HARVs. The strains were grown for 7 h in the HARVs at 37°C and 25 rpm unless noted otherwise. Cultivation for over 7 h in the HARVs leads to substantial cell lysis (data not shown). Therefore, all secretion-related experiments were completed within 7 h to minimize experimental complications from cellular lysis. The cultures were centrifuged at 4,000 x g for 10 min to separate the cells from the media. The supernatant is considered the secretion fraction. Sodium dodecyl sulfate-polyacrylamide electrophoresis (SDS-PAGE) samples for the secretion fractions were prepared by adding Laemmli buffer to the supernatant. These were compared to whole culture lysate samples, which were not subjected to centrifugation. Instead, for whole culture samples, the cell cultures were mixed directly with Laemmli buffer. All SDS-PAGE samples (secreted fraction and whole culture) were prepared by boiling at 95°C for 5 min immediately after sample preparation, which lyses the whole culture samples. The samples were loaded onto 12.5% SDS-PAGE gels and electrophoresed at 130 V for 70 min. After the separation of each sample by SDS-PAGE, proteins were transferred to a polyvinylidene difluoride (PVDF) membrane (Millipore) for Western blotting. Membranes were probed overnight at 4°C with mouse anti-FLAG (Sigma-Aldrich F3165) or anti-GroEL (Sigma-Aldrich G6532) antibodies according to the manufacturer’s protocol. Briefly, the antibodies were diluted in TBST buffer (2.42 g/L Tris base, 8.76 g/L of NaCl, 0.05% v/v Tween-20, pH 7.5) at ratios of 1:6666 and 1:10000, respectively. Chemiluminescent detection was carried out with secondary antibodies for 1 h at room temperature using goat anti-mouse IgG (Invitrogen 32430) or anti-rabbit IgG (Invitrogen 32460), each diluted 1:1000 in TBST buffer, following the manufacturer’s protocol. Western blot images were visualized using the West Pico PLUS chemiluminescent substrate (Thermo Scientific) followed by detection using a ChemiDoc XRS+ (Bio-Rad). The Western blot images, Figure 4 and Supplementary Figure 2, were cropped to exclude irrelevant regions while maintaining the integrity of this experimental data. Protein size markers were labeled in the same positions as in the original blot images. The original images were reviewed and verified during the revision process to ensure transparency. All relative protein quantities were calculated from OD_600_-normalized samples using ImageJ software and further adjusted to the average of the replicates of the specified normalization condition for relative comparison.

### 2.7 Statistical analysis

All quantitative data are presented as the mean ± standard deviation (SD) of three or four biological replicates, as noted. Statistical significance was determined using the Student’s t-test *via* Excel’s data analysis tool, with significance levels indicated as * (*p* < 0.05), ** (*p* < 0.01), and *** (*p* < 0.001).

## 3 Results

### 3.1 Establishing simulated microgravity for *S. enterica* serovar Typhimurium cultivation

**FIGURE 1:**
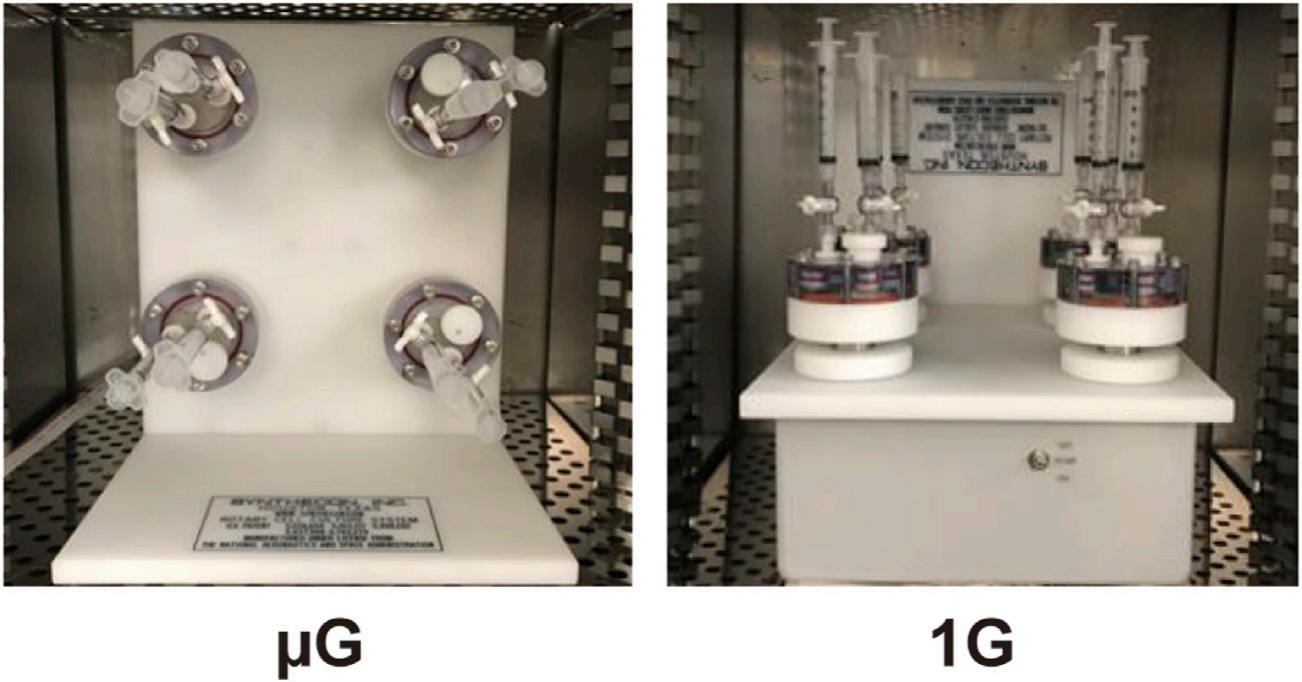
Setup of Rotary Cell Culture System (RCCS) with High Aspect Ratio Vessels (HARVs) to generate simulated microgravity (µG, left) and Earth gravity (1G, right) conditions.

The RCCS is a bioreactor system that provides simulated microgravity and low-shear environments (<1 dyn/cm^2^) for microbial culture by rotating the culture vessels at speeds to avoid cell sedimentation (Nickerson et al., 2003; Anken, 2013; Acres et al., 2021). In order to investigate the impact of simulated microgravity on our *S*. *enterica* ASTE13 protein production strain, we needed to establish a protocol for bacterial culture and sampling in the system under simulated microgravity that was amenable to adaptation for Earth gravity operation as a control. Briefly, the RCCS is designed to be operated in the simulated microgravity (vertical) orientation, such that rotation of the HARVs is in the plane vertical to the ground and leads to perpetually falling cells within the culture (Figure 1). As a control orientation, we chose to run the system in Earth gravity (horizontal) orientation. The RCCS is not designed to operate in this condition, so we used a custom-fabricated support in order to hold the RCCS in this Earth gravity orientation, which allowed rotation in the plane horizontal with the ground. The RCCS with HARVs was placed in the humidified incubator to avoid media evaporation. We selected a 25 rpm rotation speed for the HARVs regardless of orientation, because this speed emulates microgravity environments in this system when operated with rotation in the vertical orientation (Nickerson et al., 2000; Wilson et al., 2002; Crabbé et al., 2010; Yang et al., 2016; Kim and Rhee, 2016; Kim and Rhee, 2021). Importantly, the RCCS with HARVs at this speed avoids fluid mixing as assessed with a dye experiment (Crabbé et al., 2010).

Sampling from the HARVs during cell culture requires using a syringe and the sample port (Supplementary Figure 1), which must be done while rotating the vessels. The procedure is challenging in the microgravity orientation, and would alter vessel volume, potentially causing unknown effects on the simulated microgravity environment. Importantly, this process also does not yield homogeneous samples at Earth gravity due to cell sedimentation. To avoid these two critical issues, we opted to use only end-point sampling. The resulting procedure requires more time and samples (one HARV culture is used per time point), but we reasoned it should lead to more reproducible datasets.

### 3.2 Simulated microgravity enhances stress tolerance in *S. enterica* serovar Typhimurium ASTE13

**FIGURE 2:**
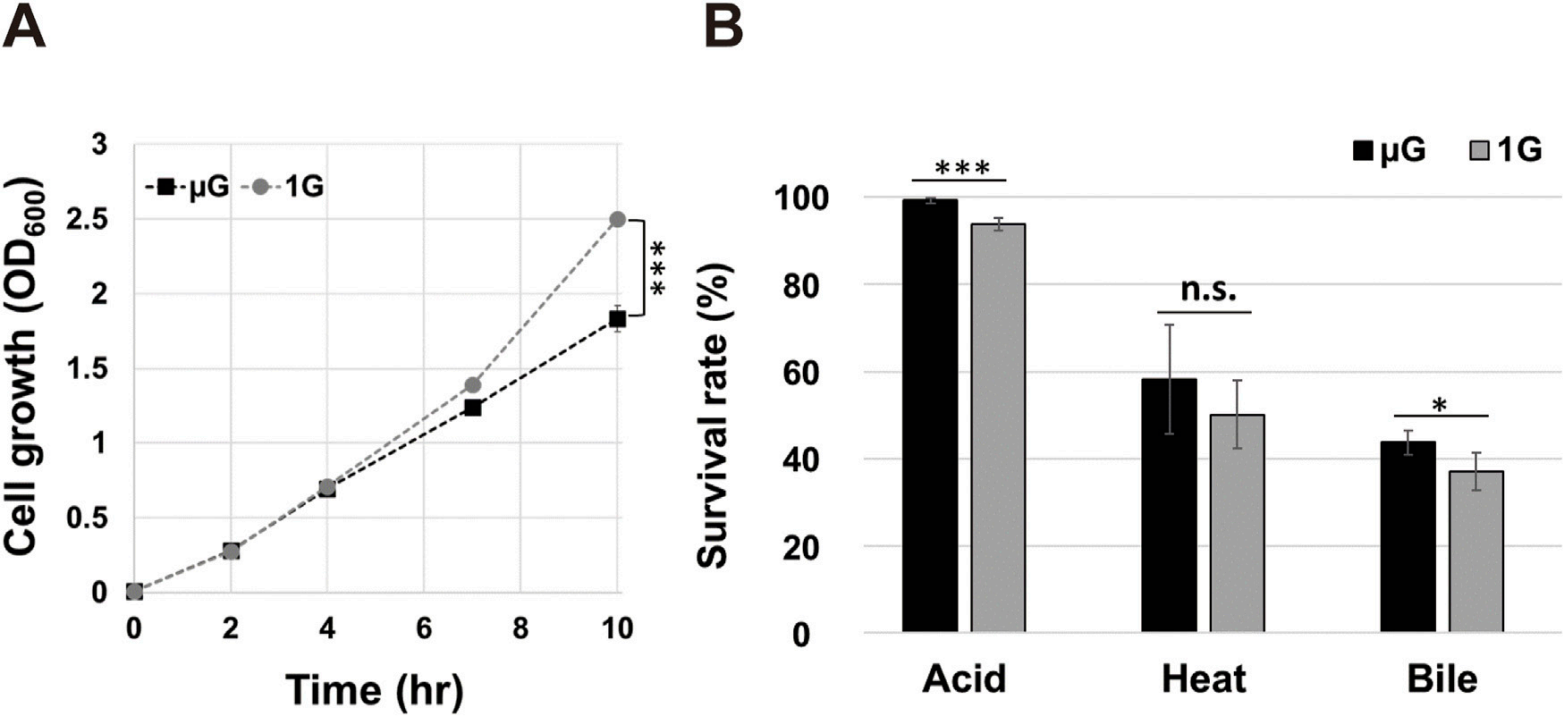
Comparison of *Salmonella* ASTE13 growth and stress resistance under simulated microgravity (µG) and Earth gravity (1G) conditions. **(A)** Growth of the *Salmonella* ASTE13 strain cultured under µG and 1G was measured by optical density at 600 nm (OD_600_) after end-point sampling. Data represent the mean of three biological replicates, with error bars displaying standard deviation. **(B)** Stress resistance of *Salmonella* ASTE13 strain cultured under µG and 1G. After 10 h of growth in HARVs with RCCS, the strain was exposed to acid stress (pH < 4), heat stress (55 °C), and bile stress (10% bile salts) for 30 min. Survival rates were determined using the LIVE/DEAD Cell Viability Assays. Data represent the mean of three biological replicates with error bars indicating standard deviation. A two-tailed Student’s t-test was performed to determine a statistically significant difference between two gravitational conditions. The significances were marked as *** (*p* < 0.001), * (*p* < 0.05), and n. s. (*p* > 0.05).

Using the RCCS bioreactor culture system established above, we next set out to determine whether gravity alters cell growth and stress responses for ASTE13. We cultured the bacteria for 10 h in LB media in both microgravity and Earth gravity environments, taking time points throughout the culture period. Cell growth appears indistinguishable between the two conditions through the 4-h time points, but then begins to deviate. At 10 hours, a 36% growth enhancement (*p* < 0.001) was observed in the strain grown in the Earth gravity orientation as compared to the microgravity orientation (Figure 2A).

*Salmonella* stress tolerance is impacted by growth under microgravity as opposed to Earth gravity conditions, but the effects can vary depending on strain, nutrition, and other culture conditions. For example, *S*. *enterica x3339* and *x4973* exhibited enhanced stress tolerance to low pH, heat, and osmotic shock when grown under simulated microgravity compared to Earth gravity (Nickerson et al., 2000; Wilson et al., 2002), while *S*. *enterica* D23580 decreases in stress tolerance to either low pH or bile stress following a simulated microgravity exposure (Yang et al., 2016). Likewise, studies on other microbial strains have reported varying stress responses to environmental factors such as low pH, low and high temperature under different gravitational conditions (Crabbé et al., 2010; Kim and Rhee, 2016; Sheet et al., 2020; Kim and Rhee, 2021). Therefore, we sought to examine the stress responses of our *S. enterica* serovar Typhimurium ASTE13 strain, specifically targeting low pH, heat, and bile salt as stress stimulators─conditions known to elicit marked stress responses in *Salmonella* (Humphrey, 2004; Prieto et al., 2004). We grew ASTE13 under both simulated microgravity and Earth gravity conditions, and then exposed each sample to environmental stressors. Stress resistance was assessed by determining survival rates using a viability assay after 30 min of exposure to each stressor. The cells grown at simulated microgravity exhibited higher survival rates for both acid stress (*p* < 0.001) and bile salts (*p* < 0.05) compared to those grown at Earth gravity (Figure 2B) while survival for heat stress was not significantly different across gravity environments.

### 3.3 The *sic* operon of the SPI-1 T3SS is transcriptionally active in simulated microgravity

**FIGURE 3:**
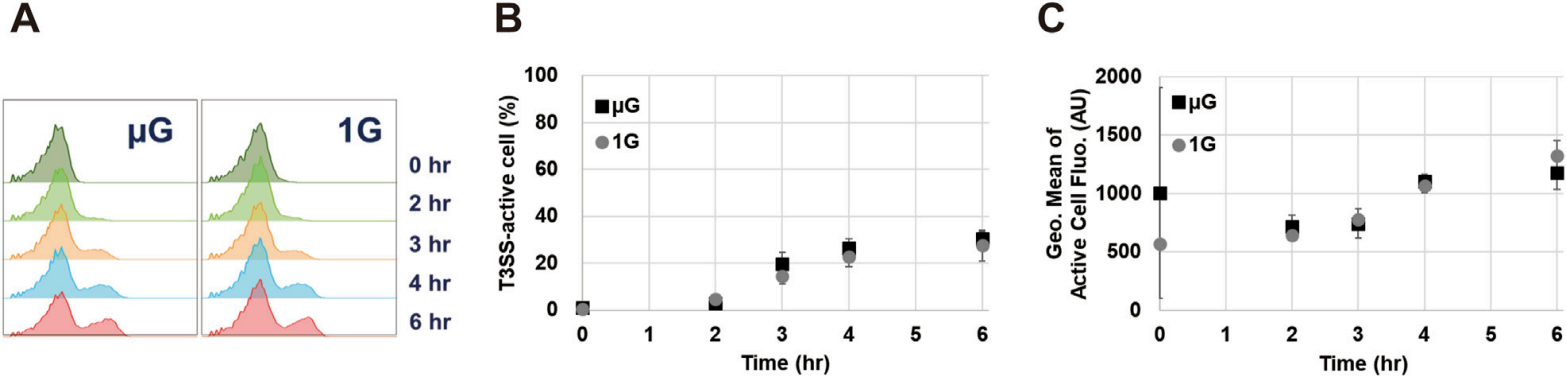
Comparison of SPI-1 T3SS transcriptional activity under simulated microgravity (µG) and Earth gravity (1G) conditions. **(A)** Representative histograms showing the GFP signal detected *via* flow cytometry in *sipC*:GFPmut2 indicating transcriptional activity from *sicA* promoter. Histograms are representative of three to four biological replicates. **(B)** percentage of cells expressing GFP signal, and **(C)** geometric mean fluorescence intensity of the GFP signal representing T3SS activity. Data in B and C shows the mean value of three to four biological replicates with error bars indicating standard deviation. Measured values for simulated microgravity were not significantly different (*p* > 0.05) to those observed at Earth gravity at any time point.

Slight environmental changes can alter the SPI-1 T3SS transcriptional activity, in turn affecting protein secretion titer and efficiency (Tartera and Metcalf, 1993; Burdette et al., 2021; Liang et al., 2023). Given the distinct impact of gravity on stress responses, we wished to investigate whether gravity influences the transcriptional activity of SPI-1 T3SS. For this purpose, we used a variation of the ASTE13 strain harboring a GFP reporter encoded in place of the downstream SPI-1 effector *sipC* (*sipC*:GFPmut2), which reports on the activity of the *sic* promoter (Burdette et al., 2021). The *sic* promoter is activated when the T3SS needle is constructed and operational (Darwin and Miller, 2000). SipC, known as a needle tip complex protein, is located within the *sic* operon, and its expression is controlled by the *sic* promoter (Lou et al., 2019). The *sic* promoter plays a critical role in the secretion-activated network of SPI-1 T3SS operon, driving the expression of genes involved in protein secretion (Widmaier et al., 2009). The promoter is the final promoter to be activated in the secretion activation cascade. Given that our goal is to establish active secretion through the SPI-1 T3SS under space conditions, we reasoned that this promoter is an excellent proxy for the full activation of the T3SS. Using the strain harboring the GFP reporter, we examined *sic* promoter activity by monitoring GFP fluorescence on a per-cell basis using flow cytometry over time from 0 to 6 h (Figure 3). An active SPI-1 T3SS population was detected in 3-h samples for both gravity environments (Figures 3A, B). This population increases in both size of the “*sic* active” population (Figure 3B) and the fluorescence intensity of this fraction of the population (Figure 3C). Importantly, T3SS activation under simulated microgravity was not significantly different (*p* > 0.05) to that observed at Earth gravity at all time points. These results are encouraging for the potential use of the SPI-1 T3SS as a protein production platform in space.

### 3.4 Protein secretion *via* SPI-1 T3SS occurs in simulated microgravity

**FIGURE 4:**
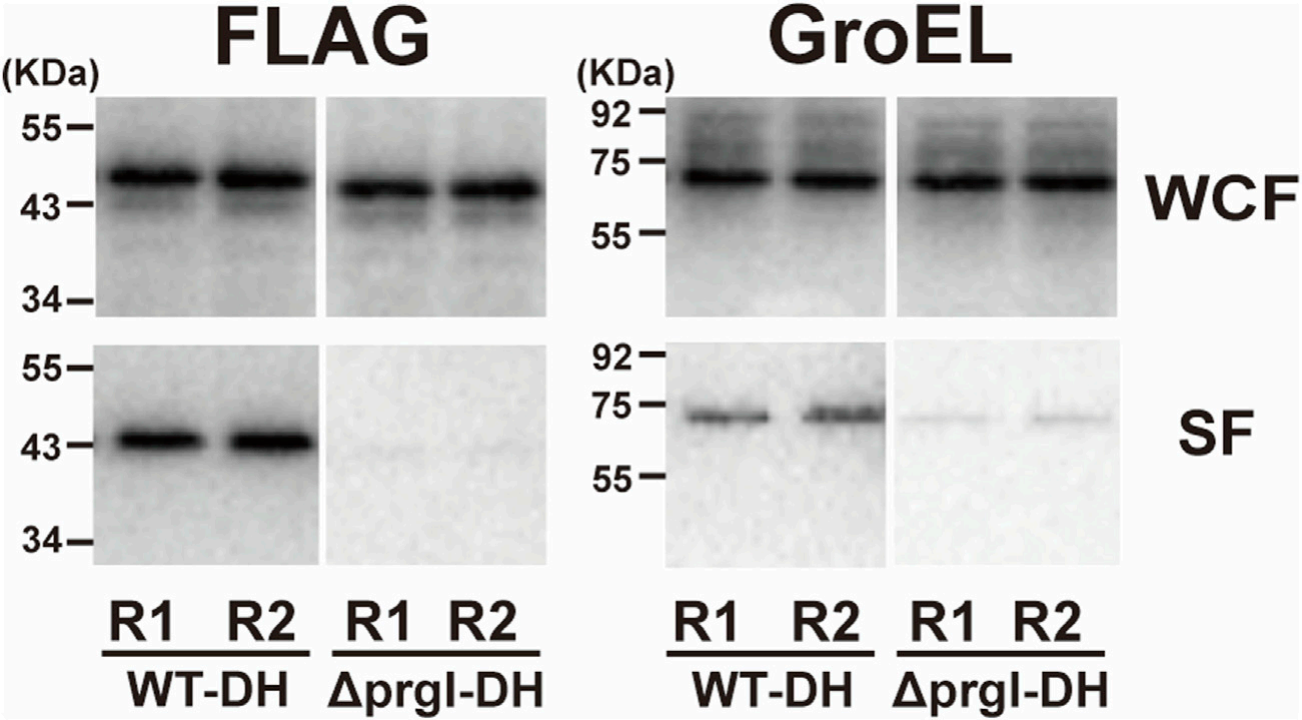
Evaluation of DH protein secretion through SPI-1 T3SS in simulated microgravity (µG) conditions. The WT-DH and Δ*prgI*-DH strains were cultured for 7 h at 25 rpm in HARVs with RCCS. Protein secretion was analyzed by Western blotting, and two biological replicates (marked as R1 and R2) from WT-DH and Δ*prgI*-DH strain were loaded on SDS-PAGE gel. Loading volumes were determined by OD_600_. SF and WCF represent the secreted fraction and whole culture lysate fraction, respectively.

We next directly evaluated the protein secretion capabilities of the SPI-1 T3SS in our lab-developed ASTE13 strain using the ground-based bioreactor system. To assess protein secretion into the extracellular environment, we transformed ASTE13 with an export plasmid, which harbors a gene encoding the DH protein fused to an N-terminal SptP secretion sequence and a C-terminal FLAG tag, all under the control of the *sic* promoter (Widmaier et al., 2009). The DH protein is the 24 kDa catalytic domain of the human intersectin-1 protein that has long served as a model secretion substrate for the SPI-1 system, with SptP as well as other N-terminal secretion tags (Widmaier et al., 2009; Metcalf et al., 2014; Burdette et al., 2021; Liang et al., 2023). Using the DH protein, therefore, enables the assessment of secretion efficiency in the context of the established SPI-1 secretion model and in comparison to prior studies. Concurrently, to help assess whether DH protein detected in the supernatant is the result of SPI-1 T3SS-mediated protein secretion, we used another strain, ASTE13 Δ*prgI*, to serve as a negative control (Metcalf et al., 2014; Burdette et al., 2024). This strain contains a deletion of the gene encoding the needle-forming protein PrgI, a key SPI-1 T3SS component. In the absence of PrgI, the needle structure is not formed, and the strain cannot secrete proteins *via* SPI-1 T3SS (Metcalf et al., 2014; Metcalf et al., 2016; Burdette et al., 2024). We used Western blotting against the FLAG tag to assess protein expression and secretion for both ASTE13 and our negative control ASTE13 Δ*prgI* strain. To assess protein expression, we examined protein content of the entire culture (lysed bacterial cells plus the media), while to assess protein secretion, we examined the media of the culture alone by pelleting the bacterial cells. Under the simulated microgravity condition, DH protein expression levels were similar in both strains. However, the media of the ASTE13 strain exhibited intense protein bands while the non-secreting negative control strain had no detectable protein bands at the size expected for the DH fusion under the simulated microgravity condition (Figure 4; Supplementary Figure 2). From the data, we concluded that the DH protein is secreted in a T3SS-dependent manner under simulated microgravity.

We next set out to explore the secretion efficiency of SPI-1 T3SS under simulated microgravity and Earth gravity conditions. In both gravity conditions, similar levels of DH protein expression were observed (Supplementary Figure 3). The strains grown at Earth gravity exhibited 2.3-fold (*p* < 0.001) more intense DH protein bands in the secreted fraction than those grown under simulated microgravity (Supplementary Figure 3). However, intracellular GroEL protein bands were also approximately 1.5-fold (*p* < 0.05) higher in the secreted fraction under Earth gravity conditions. In contrast, the GroEL protein bands under simulated microgravity were detectable but displayed relatively low intensity. Taken together, our results show there is measurable cell lysis in the HARV format, precluding comparison of secretion efficiency across gravity conditions (Supplementary Figure 3).

## 4 Discussion

With the expansion of the space economy, interest in space exploration and opportunities continue to grow (Corrado et al., 2023). However, astronauts face health and safety challenges during missions, particularly due to the resource-limited environments (Braddock, 2017; Douglas et al., 2020; Douglas et al., 2021). Therefore, there is an increasing need for robust systems that provide essential goods such as medicine, food, and energy through ISRU (Jemison and Olabisi, 2021; Berliner et al., 2024). The advancements in biotechnology have led to the development of microorganisms as efficient cell factories for producing useful chemicals and proteins owing to their rapid growth and ease of genetic manipulation (Menezes et al., 2015; Nielsen and Keasling, 2016; Sanchez-Garcia et al., 2016; Santomartino et al., 2023). In principle, by maintaining bacterial stocks and a DNA synthesizer, a variety of bioproducts could be produced on demand from the same reagents. Despite this potential, space environments impose unique constraints on microbial systems, impacting their physiological, metabolic, and genetic behavior compared to what may be observed on Earth. To fully leverage microorganisms for space applications, we must understand how space conditions affect biological systems. Several prior studies suggested that simulated microgravity can enhance protein production (Xiang et al., 2010; Huangfu et al., 2020; Chen et al., 2021). Here, we examined a potential method for facile protein purification to utilize this increased protein production in a space or resource-limited environment.

For this purpose, we assessed *S. enterica* serovar Typhimurium for its potential as a protein production bio-factory. Since *S. enterica* includes pathogenic strains, its beneficial applications in biotechnology are often overlooked. However, some *S. enterica* strains have been successfully utilized as vaccines (e.g., Ty21a and Vi CPS) (MacLennan et al., 2014) and are also considered a promising vehicle for the delivery of therapeutic proteins for anti-cancer drugs and immunotherapies, with approval from the U.S. Food and Drug Administration (Badie et al., 2021; Aganja et al., 2022). In particular, we explored the robustness of the SPI-1 T3SS, an efficient protein secretion system found in *S. enterica*, using ground-based, microgravity-simulating bioreactors. The SPI-1 T3SS offers a simplified and efficient method for extracellular recombinant protein production bypassing the need for complex downstream processing. However, its performance under microgravity has not been thoroughly evaluated. Nickerson and coworkers reported increased virulence of *S. enterica* grown in microgravity and simulated microgravity environments (Nickerson et al., 2000). Because the T3SS encoded by SPI-1 is natively an important factor for *S. enterica* virulence in pathogenic strains (Green and Mecsas, 2016; Burdette et al., 2018; Lou et al., 2019), we reasoned that the SPI-1 T3SS may maintain or even increase in activity under microgravity conditions, and therefore be useful as a platform for producing recombinant proteins such as therapeutic agents and enzymes in space *via* ISRU.

Our laboratory strain *S. enterica* ASTE13 provides high growth and protein secretion and was engineered for industrial protein production applications. Our results indicate that ASTE13 responds to microgravity by exhibiting altered growth and stress responses (Figure 1). The reduced growth observed in this study may be attributed to a stress response triggered by simulated microgravity. Previous research has demonstrated that microgravity can act as a stress stimulus in *S. enterica* (Nickerson et al., 2000; Wilson et al., 2002), resulting in altered cellular responses. Similarly, a recent study in *E. coli* reported not only growth reduction under simulated microgravity condition but also the upregulation of stress-related genes compared to the Earth gravity condition (Yim et al., 2020). When we compared the stress tolerance under both gravitational conditions, the heat stress resistance of ASTE13 under simulated microgravity was not as significant as the acid and bile-induced stresses. *Salmonella* employ a range of distinct mechanisms to counteract diverse stressors (Shen and Fang, 2012). Acid and bile stress resistance frequently involves the activity of efflux pumps or modifications to the outer membrane (Gunn, 2000; Schumacher et al., 2023), while heat shock resistance primarily relies on the synthesis of heat shock or chaperone proteins (Roncarati and Scarlato, 2017). The activation of stress responses under simulated microgravity may have enhanced resistance to acid and bile stress but did not sufficiently prepare the cells for heat shock resistance. This indicates that ASTE13 grown under simulated microgravity might not have been physiologically adapted to tolerate heat stress as readily as acid and bile-induced stresses. The increased stress tolerance observed in ASTE13 under simulated microgravity can be considered a desirable feature for its use in space environments.

Importantly, the microgravity condition does not appear to negatively impact the functionality of SPI-1 T3SS. Both protein expression levels and transcriptional activity remained comparable to those observed at Earth gravity (Figure 3; Supplementary Figure 3). Furthermore, our experiment with the ASTE13 Δ*prgI* confirmed that *S. enterica* could efficiently secrete proteins through the SPI-1 T3SS even under microgravity conditions (Figure 4; Supplementary Figure 2). While this is useful in our ASTE13 strain for protein production, it raises questions with respect to how virulent strains may behave. However, we note that evolution optimized secretion levels for infection, so if the observed increases in ASTE13 extend to more pathogenic strains, this still may not translate to increased virulence. Additional studies are warranted to explore the impact of microgravity on pathogenicity.

The RCCS with HARVs as a microgravity simulator may not be ideal for cultures requiring extended incubation times due to the potential of biofilm formation (Diaz et al., 2022) and the cell lysis we observed in this study. Since protein secretion *via* the T3SS of ASTE13 has not given rise to extensive cell lysis when grown with shaking in the standard laboratory environment, and the observed effects were less under the microgravity condition than the Earth gravity condition, we conclude that something related to the HARV format (e.g.*,* changes in oxygen delivery) is responsible for inducing lysis. For all bacterial culture studies using the HARV system, it will be imperative to understand and address cell lysis in advance. However, since the *S. enterica* T3SS protein production platform requires only short culture durations, this microgravity-simulating bioreactor system provides an efficient test platform for studying microgravity impacts on the T3SS. To further reduce lysis, future studies on this system could further optimize the culture conditions in microgravity-simulating bioreactor systems or engineer ASTE13 to enhance its robustness, e.g.*, via* adaptive laboratory evolution using growth in the HARV.

Overall, our findings suggest that the SPI-1 T3SS is still active in microgravity, making it a promising platform for protein production in space. To further refine this system for space applications, we propose integrating systems biology approaches to identify the engineering targets that could optimize the SPI-1 T3SS for efficient protein secretion machinery in space. Additionally, it would be useful to evaluate the versatility of *Salmonella* T3SS under the two distinct gravitational conditions by introducing a range of distinct protein classes as secretion substrates, such as enzymes, potential receptor-binding proteins, and material-forming proteins. Such experiments will serve to further optimize the capacity of the system, particularly with regard to the folding, functionality, and activity of secreted proteins. Finally, a complete biosafety evaluation of *S. enterica* ASTE13 is warranted given its potential as a protein producer and its close evolutionary relationship to more pathogenic strains. Should such studies reveal potential for latent pathogenicity, strain engineering could be used to address such issues. Engineering of ASTE13 to limit pathogenicity and enhance protein production in a standard laboratory environment as well as under simulated microgravity will inform engineering strategies for future space missions.

## Data Availability

The original contributions presented in the study are included in the article/Supplementary Material, further inquiries can be directed to the corresponding author.
