# Supplementary figures and images for: Evaluation of the Salmonella type 3 secretion system (T3SS) as part of a protein production platform for space biology applications
Source: Front Bioeng Biotechnol. 2025 Apr 2;13:1567596. doi: 10.3389/fbioe.2025.1567596 (PMC12000002; doi:10.3389/fbioe.2025.1567596)

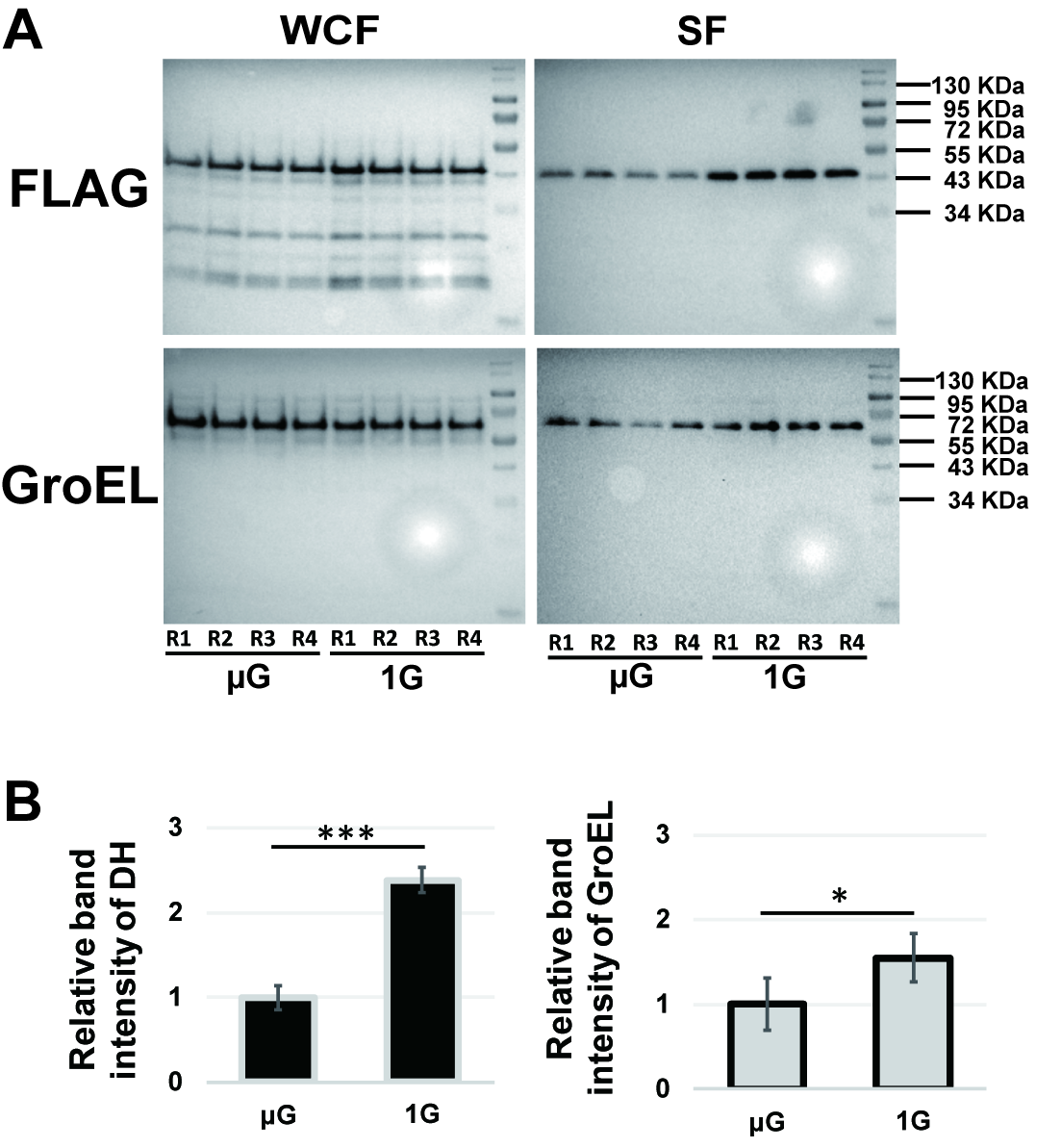

Supplement: Supplementary file 1 [file Image3.tif]

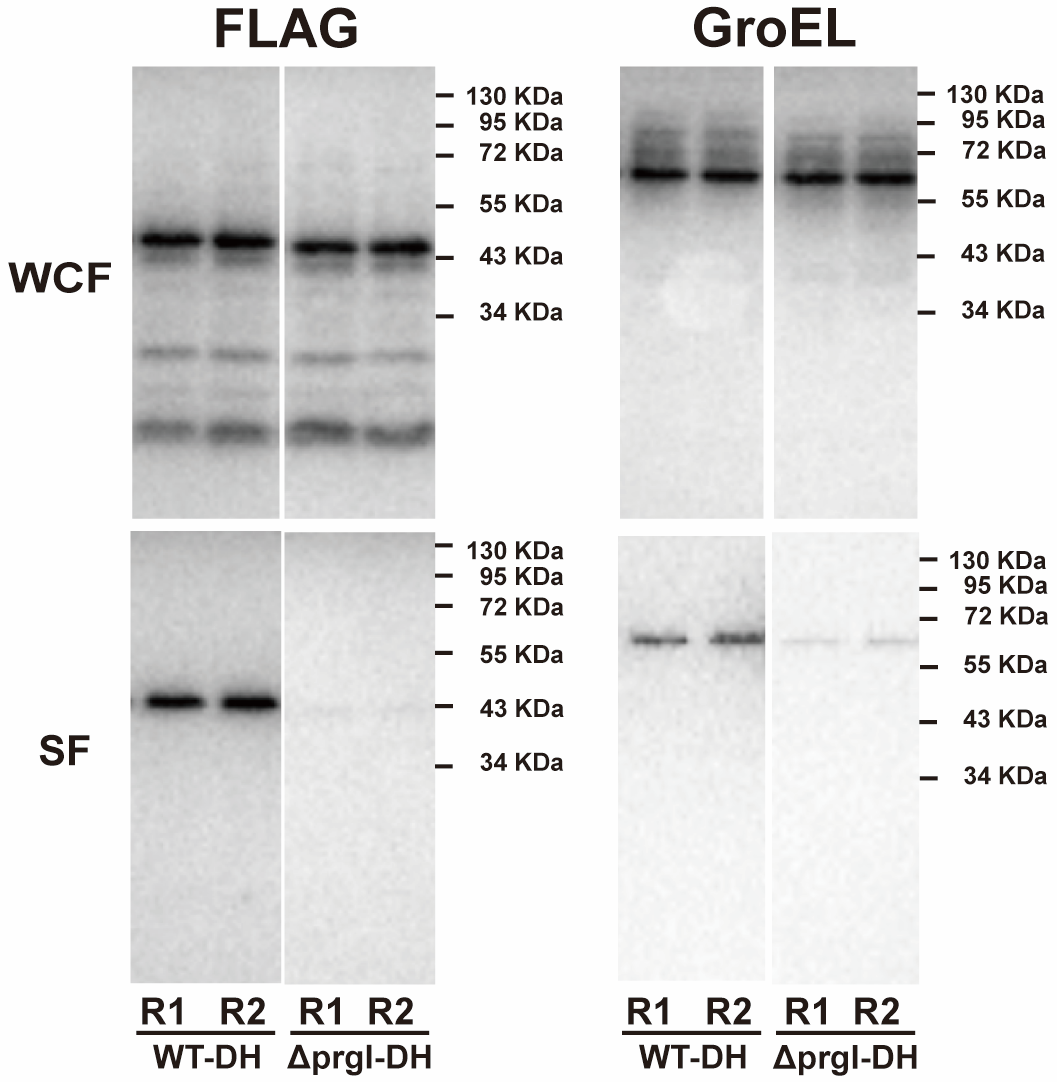

Supplement: Supplementary file 2 [file Image2.tif]

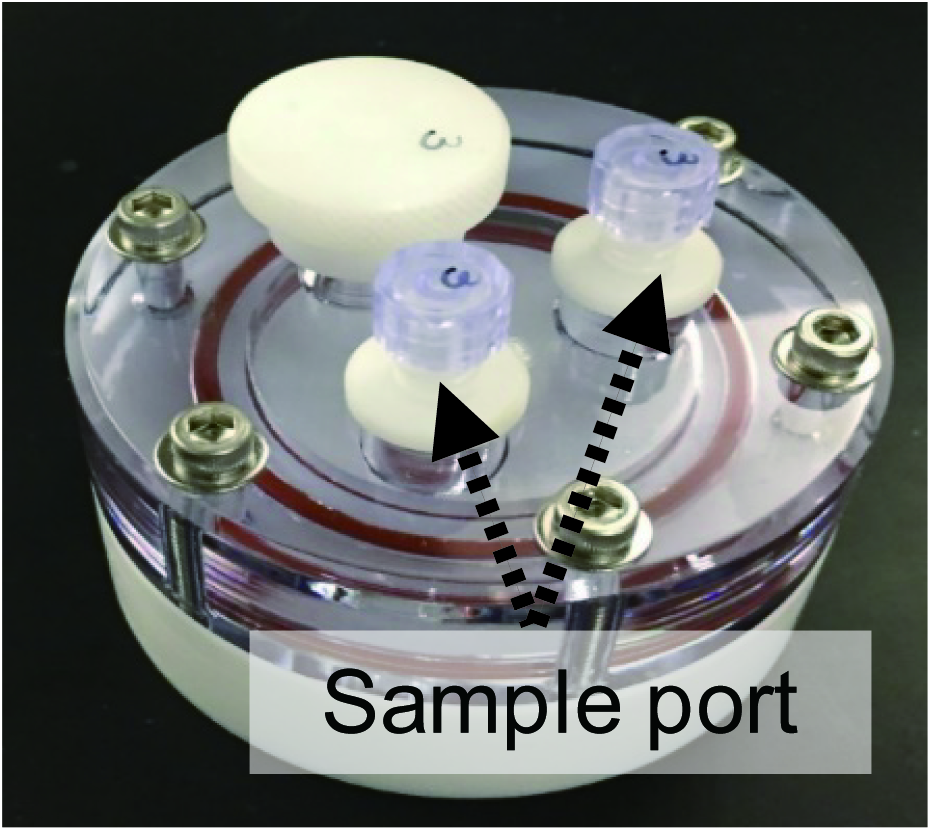

Supplement: Supplementary file 3 [file Image1.tif]
