# Supplementary material for: Evaluation of the Salmonella type 3 secretion system (T3SS) as part of a protein production platform for space biology applications
Source: Front Bioeng Biotechnol. 2025 Apr 2;13:1567596. doi: 10.3389/fbioe.2025.1567596 (PMC12000002; doi:10.3389/fbioe.2025.1567596)
Supplement: Supplementary file 4 [file DataSheet1.docx]

Supplementary Material

**Evaluation of Salmonella type 3 secretion system (T3SS) as part of a**

**protein production platform for space biology applications**

Min-Kyoung Kang, James Bevington, Danielle Tullman-Ercek*

*** Correspondence:** Corresponding Author: ercek@northwestern.edu

- Supplementary Figure 1
- Supplementary Figure 2
- Supplementary Figure 3


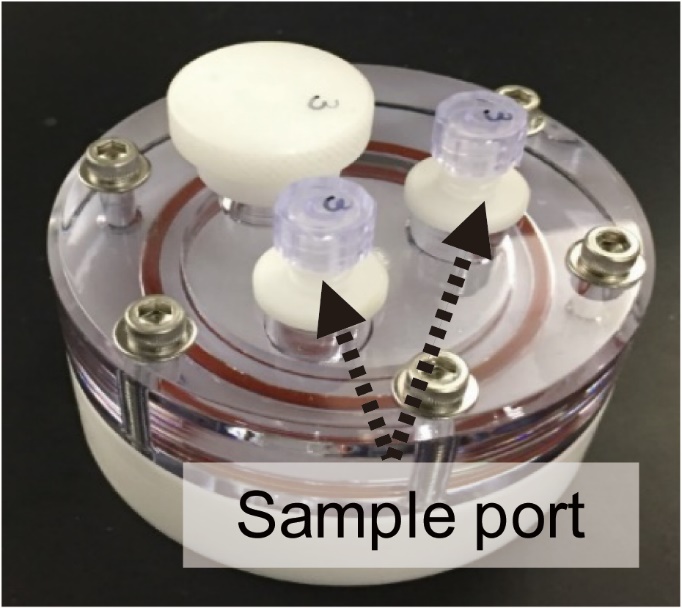


**Supplementary Figure 1.** A photo of the HARV. Arrows indicate the location of the sample port on the HARV.


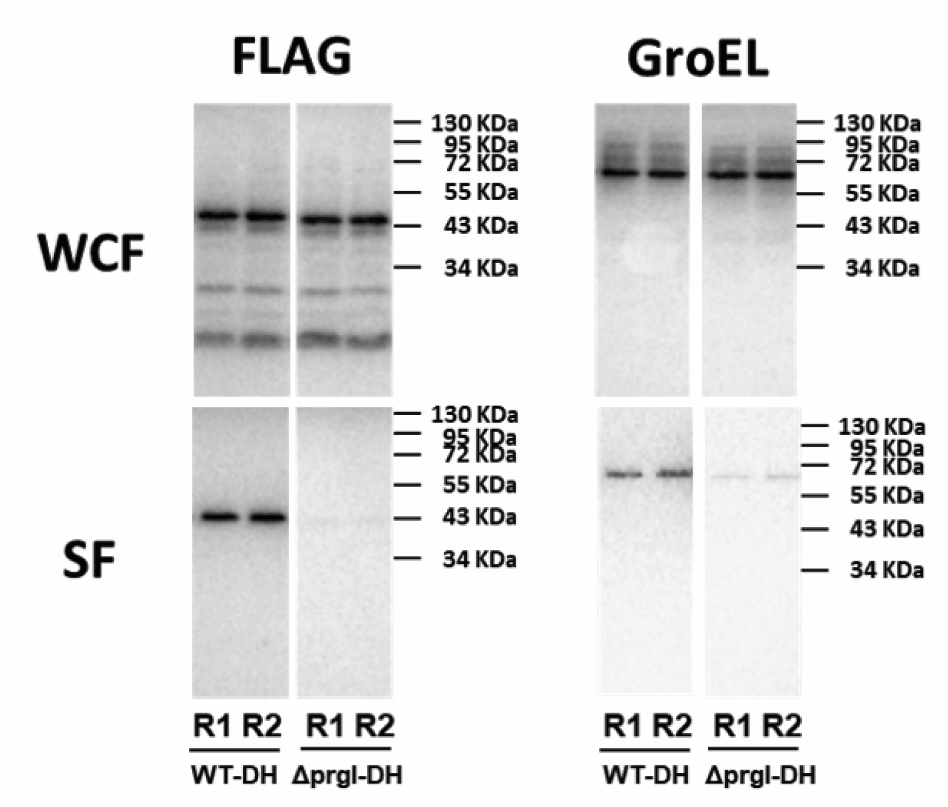


**Supplementary Figure 2.** Evaluation of DH protein secretion through SPI-1 T3SS in simulated microgravity (µG) conditions. The WT-DH and Δ*prgI*-DH strains were cultured for 7 hours at 25 rpm in HARVs with RCCS. Protein secretion was analyzed by western blotting, and two biological replicates (marked as R1 and R2) from WT-DH and Δ*prgI*-DH strain were loaded on SDS-PAGE gel. Loading volumes were determined by OD_600_. SF and WCF represent the secreted fraction and whole culture lysate fraction, respectively.


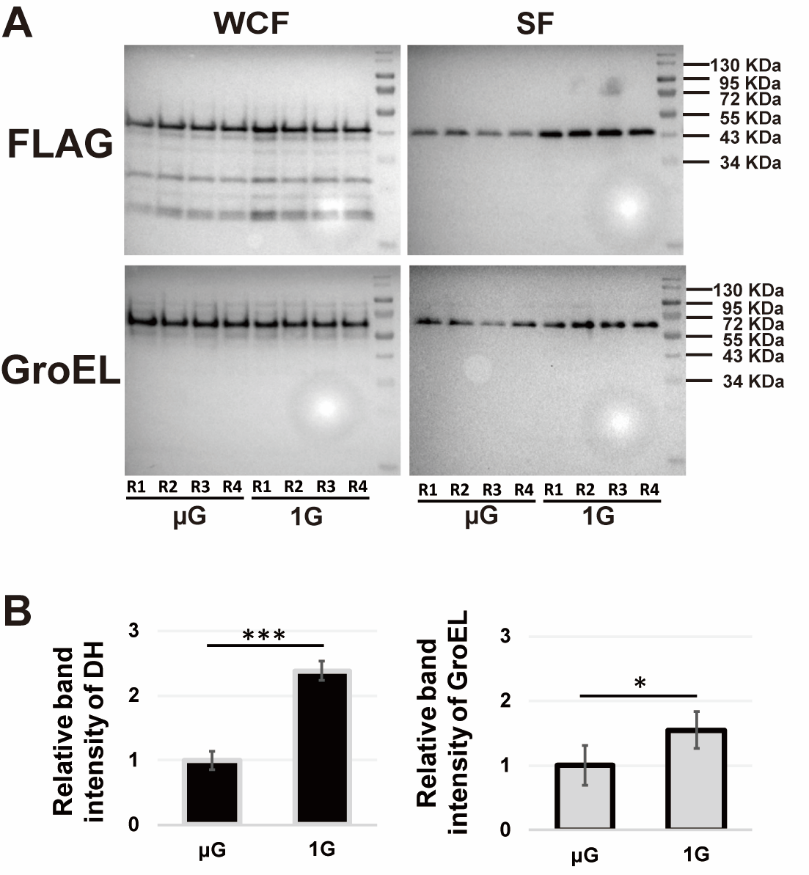


**Supplementary Figure 3.** Protein secretion through SPI-1 T3SS under simulated microgravity (µG) and Earth gravity (1G) conditions. The WT-DH strain was cultured in HARVs with RCCS for 7 hours at 25 rpm. A: Protein secretion was analyzed by western blotting with samples from four biological replicates (marked as R1-R4) per gravity condition loaded on SDS-PAGE gel. The loading volume was normalized by OD600. SF and WCF denote the secreted fraction and whole culture lysate fraction, respectively. B: The intensity of DH and GroEL bands in secreted fractions was quantified using ImageJ software. The values were obtained from four biological replicates and normalized to display the relative intensity. A two-tailed Student’s t-test was performed to determine a statistically significant difference between two gravitational conditions. The significances were marked as *** (*p*<0.001) and * (*p*<0.05).
